# Hierarchical Heterojunctions of Metal Sulfide WS_2_ Nanosheets/Metal Oxide In_2_O_3_ Nanofibers for an Efficient Detection of Formaldehyde

**DOI:** 10.3390/nano14211702

**Published:** 2024-10-24

**Authors:** Lei Zhu, Jiaxin Zhang, Jianan Wang, Jianwei Liu, Wei Yan

**Affiliations:** 1 Xi’an Key Laboratory of Solid Waste Resource Regeneration and Recycling, State Key Laboratory of Multiphase Flow Engineering, School of Energy and Power Engineering, Xi’an Jiaotong University, Xi’an 710049, China; leizhu@xjtu.edu.cn (L.Z.);; 2 School of Physics and Electrical Engineering, Weinan Normal University, Chaoyang Street, Weinan 714099, China; 3 School of Chemistry and Chemical Engineering, Xi’an University of Science & Technology, Xi’an 710054, China

**Keywords:** gas sensor, WS_2_, p-n heterojunctions, nanofibers, HCHO

## Abstract

The construction of transition metal dichalcogenides (TMDs) heterojunctions for high-performance gas sensors has garnered significant attention due to their capacity to operate at low temperatures. Herein, we realize two-dimensional (2D) WS_2_ nanosheets in situ grown on one-dimensional (1D) In_2_O_3_ nanofibers to form heterostructures for formaldehyde (HCHO) gas sensors. Capitalizing on the p-n heterojunctions formed between WS_2_ and In_2_O_3_, coupled with the high surface-to-volume ratio characteristic of 1D nanostructures, the WS_2_/In_2_O_3_ NFs sensor demonstrated an elevated gas response of 12.6 toward 100 ppm HCHO at 140 °C, surpassing the performance of the pristine In_2_O_3_ sensor by a factor of two. Meanwhile, the sensor presents remarkable repeatability, rapid response/recovery speed, and good long-term stability. The superior sensing capabilities of WS_2_/In_2_O_3_ NFs heterojunction are attributed to the combined impact of the increased charge transfer and the presence of more sites for gas adsorption. The research endows a potent approach for fabricating TMD heterojunctions to significantly enhance the gas sensing properties of gas sensors at relatively low temperatures.

## 1. Introduction

Formaldehyde (HCHO), is a harmful gas that adversely affects human respiratory health and the quality of the environment, and it mainly comes from building materials, furniture, artificial plates, and various adhesive coatings [1,2,3]. Prolonged exposure to indoor environments with elevated levels of HCHO can result in chronic health issues, including leukemia, and potentially cancer [4]. In light of these risks, the World Health Organization (WHO) has delineated a permissible safety threshold for HCHO exposure at 81 parts per billion (ppb) [5]. Consequently, it is crucial to develop a sensor for the recognition of HCHO vapors with high response values and sustained stability for indoor environmental monitoring.

Gas sensors based on metal oxide semiconductors (MOS) have garnered significant interest in the detection of HCHO [6,7,8]. Zhang et al. [9] prepared single-atom Ru-SnO_2_ nanoparticles for HCHO detection. Analyses of gas sensing performance have demonstrated that the SnO_2_/Ru sensor exhibits a high response value of 36.3 toward 20 ppm HCHO at 250 °C. Lu et al. [7] fabricated an HCHO gas sensor based on Pt_1_-In_2_O_3_ and the sensor enabled high sensitivity toward HCHO at 200 °C. Although the HCHO gas sensors prepared by the above methods have achieved certain results, and all of them show high sensitivity to HCHO gas, the traditional thermally excited MOS-based gas sensors are still required to be operated at temperatures exceeding 200 °C and have high power consumption [10,11], which can adversely impact the sensor’s service life and long-term stability. Hence, there exists demand for the advancement of HCHO gas sensors capable of functioning with low power consumption, which is conducive to the portability and miniaturization of the devices.

Two-dimensional (2D) TMDs have garnered considerable attention as potential candidates for use in gas sensing applications, owing to their enhanced specific surface area and elevated surface reactivity [12,13]. The gas sensing mechanism of TMDs relies on the charge transfer occurring on the surface of the material, i.e., the target gas molecules to be measured undergo charge transfer with the surface of the metal sulfide material through electrostatic action resulting in modifications in the sensor’s total electrical conductivity [14,15]. Since TMDs have a narrow bandgap, gas molecules can undergo adsorption and desorption reactions on the surface of the material at low or even room temperatures [16]. Among distinctive monolayer TMDs, tungsten sulfide (WS_2_) has unique properties including good thermal stability, cost-effectiveness, and adjustable energy band structure. Numerous investigations have been conducted on the utilization of WS_2_ for gas detection at low or room temperatures [17,18,19,20]. Despite these efforts, there remains a necessity for further enhancements in the areas of accelerating the response and recovery times, and in improving the long-term stability of WS_2_ gas sensors. To address the above problems, the hybridization of MOS with WS_2_ has been proposed for regulating the Fermi energy levels within the gas sensing materials and altering their inherent charge transport mechanisms, thereby significantly enhancing the overall performance of the gas sensing [21,22,23]. For example, Qin et al. [21] reported the improved sensing performance of TiO_2_ quantum dots (QDs) decorated WS_2_ nanohybrids in detecting NH_3_ at room temperature due to the effective electron transfer between the WS_2_ and the TiO_2_ QDs through S-O-Ti bonding. Kim et al. [24] found that the WS_2_-ZnO heterojunction sensor exhibited the highest response to reducing gas under a low applied voltage of 4.2 eV.

In this work, one-dimensional (1D) In_2_O_3_ nanofibers (NFs) were selected as the foundational phase for the in situ growing of 2D WS_2_ nanosheets, thereby constructing a heterojunction structure. These heterojunctions offer a close contact surface and robust chemical bonds, which in turn boosts the carrier density and facilitates charge transfer. The WS_2_/In_2_O_3_ heterostructures sensor enables a notable response value of 12.6 exposure to 100 ppm HCHO at 140 °C, along with rapid response/recovery time (30/43 s). In addition to these characteristics, the device has shown satisfactory repeatability and sustained stability over time. To further elucidate the enhanced sensing mechanism for HCHO detection, a thorough analysis of the experimental data has been conducted on the WS_2_/In_2_O_3_ sensor.

## 2. Experimental Section

### 2.1. Synthesis of Pristine In_2_O_3_ Nanofibers (NFs)

The synthesis of pristine indium oxide nanofibers (In_2_O_3_ NFs) was achieved through a process involving electrospinning followed by a subsequent annealing procedure. Typically, 1.0 g In(NO_3_)_3_·4.5H_2_O was introduced into a blend consisting of 10 mL N, N-dimethylformamide, and 4 mL ethanol and stirred for 2 h. Then, 1.0 g polyvinylpyrrolidone (PVP) was uniformly dispersed into the mixture and stirred continuously for an additional 4 h. The resulting homogeneous electrospinning solution was subsequently loaded into a 5 mL syringe. The spinning process was then carried out under the influence of an electrostatic field with a voltage of approximately 14 kV. Following this, the precursor fibers underwent calcination at a temperature of 600 °C with a controlled heating rate of 5 °C·min^−1^ for 3 h in air, leading to the formation of the pristine In_2_O_3_ NFs.

### 2.2. Synthesis of WS_2_/In_2_O_3_ Nanofibers (NFs)

**Figure 1:**
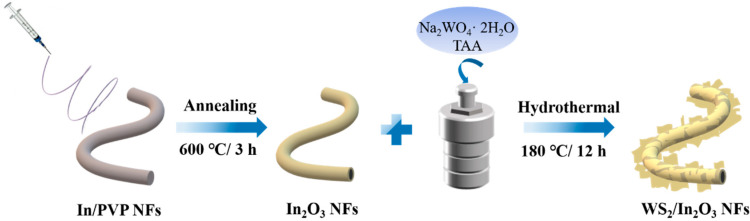
Schematic illustrations of the fabrication of WS_2_/In_2_O_3_ composite nanofibers.

The sensing materials of the WS_2_/In_2_O_3_ NFs were synthesized by a hydrothermal approach, utilizing sodium tungstate dihydrate (Na_2_WO_4_·2H_2_O) and thioacetamide (TAA) as precursors for tungsten and sulfur, respectively, as depicted in Figure 1. Specifically, 0.032 g Na_2_WO_4_·2H_2_O and 0.0751 g TAA were thoroughly mixed with deionized water (30 mL) with magnetic stirring for 30 min. Next, 0.02 g oxalic acid and 0.05 g as-prepared In_2_O_3_ NFs were introduced into the solution, which was then stirred continuously for an additional hour to ensure homogeneity. The resulting solution was then transferred into a 50 mL Teflon reactor and maintained at a temperature of 180 °C for 12 h. After the centrifugation process, the precipitate was carefully collected and thoroughly washed using ethanol to ensure purity. Subsequently, the resulting WS_2_/In_2_O_3_ nanofibers (NFs) were subjected to a drying process at 60 °C overnight. For comparison, WS_2_ nanosheets (NTs) were also synthesized via an identical procedure, except for the In_2_O_3_ NFs addition.

### 2.3. Characterizations

Morphological data of the samples were obtained using SEM (GeminiSEM 500, China) and TEM (JEOL JEM2100, Tokyo, Japan). The crystallographic information of samples was determined using X-ray diffraction (XRD) analysis (PANalytical X’pert MPDPro system, Almelo, The Netherlands) with Cu Kα radiation source operating at 40 kV and 40 mA. For the acquisition of X-ray photoelectron spectroscopy (XPS) data, the AXIS ULtrabld (UK) instrument was employed. This device used a monochromatic Al Kα radiation source with settings of 15 kV and an energy of 1486.6 electron volts. The structure and chemical bonds were determined using Fourier-transform Infrared Spectroscopy (Bruker Tensor 37 spectrometer, Billerica, MA, USA). The Raman scattering analysis was performed using an HR800 Raman spectrometer (France). For the excitation process in this analysis, a laser light with a wavelength of 532 nanometers was utilized. The surface area and pore size were measured using a Brunauer–Emmett–Teller (BET) analyzer (SSA-4300, China). The measurements were conducted at a temperature maintained by liquid nitrogen (77 K). Thermogravimetric analysis (TGA) was performed on a TGDTG-60FTIR apparatus (China) under an air atmosphere with a heating rate of 10 °C min^−1^ from room temperature to 600 °C.

### 2.4. Device Fabrication and Gas Sensing Property Tests

The prepared sample powder was thoroughly combined with deionized water and then ground to a fine paste using an agate mortar. Next, the finely ground paste was evenly coated on the ceramic tube that had been fitted with Au electrodes and then subjected to an annealing process at 120 °C for 5 h to obtain the sensor. The performance characteristics of the sensors were assessed using a WS-30A analytical instrument. The detailed procedure is shown in the Supplementary Materials Text S1. A technique involving the static distribution of liquids was employed to generate gases of varying concentrations for testing. The evaporation apparatus is designed to ensure that a specific volume, denoted as Q, of injected liquid is rapidly transformed into the gaseous phase. The volume Q can be calculated using the formula provided (referred to as Equation (S1)). This method ensures precise control over the gas concentrations used in the experiments, which is crucial for accurate sensor performance evaluation. The sensor response to HCHO is quantified as the ratio of its electrical resistance in ambient air (denoted as R_a_) to that in test gas (denoted as R_g_), expressed as S = R_a_/R_g_.

## 3. Results and Discussion

### 3.1. Morphology Characterization and Phase Composition

**Figure 2:**
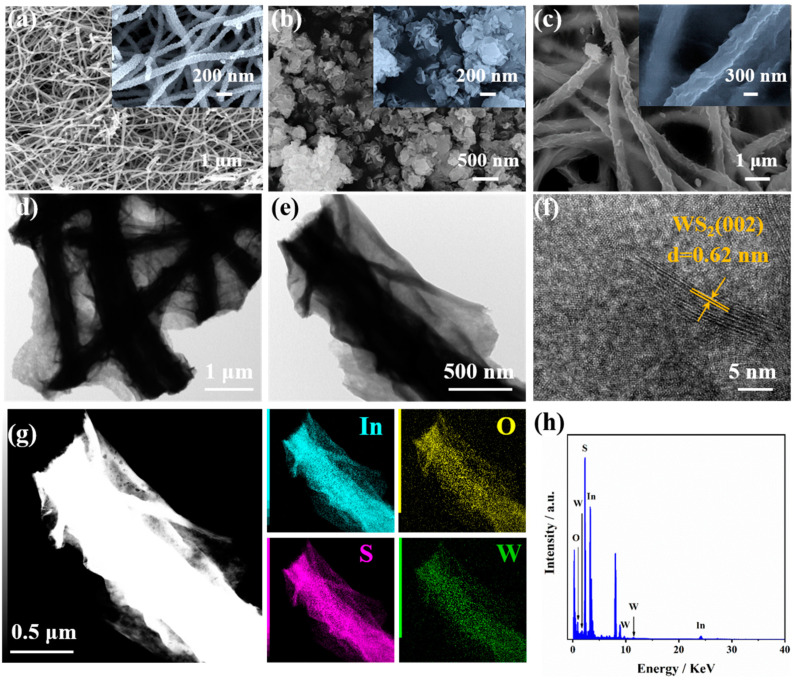
SEM images of (**a**) pristine In_2_O_3_ NFs, (**b**) WS_2_, and (**c**) WS_2_/In_2_O_3_ NFs; (**d**,**e**) TEM images, (**f**) HRTEM image, (**g**) EDS element mapping, and (**h**) EDS spectrum of WS_2_/In_2_O_3_ NFs.

The schematic illustration of the WS_2_/In_2_O_3_ NFs is illustrated in Figure 1. The microstructure and morphology of pristine In_2_O_3_ NFs, WS_2_ NTs, and WS_2_/In_2_O_3_ NFs were explored using SEM, as shown in Figure 2a–c. The pristine In_2_O_3_ NFs present a disordered fiber stacking morphology (Figure 2a). The high magnification SEM image shows that the individual fibers of In_2_O_3_ NFs are constituted by abundant uniformly sized nanoparticles (inset in Figure 2a). Figure 2b presents that the layered structure of WS_2_ is visible, appearing as a stacked nanosheet-like structure. After loading the WS_2_ on the surface of In_2_O_3_ NFs, the as-prepared WS_2_/In_2_O_3_ sample still maintains a continuous fiber morphology (Figure 2c). In addition, as shown in Figure 2c, contrasting with the pristine In_2_O_3_ nanofibers that possess a smooth surface, the surface of WS_2_/In_2_O_3_ is wrinkled due to the stacking of WS_2_ nanosheets. The average diameter of WS_2_/In_2_O_3_ NFs is increased to 0.51 μm attributed to the loading of WS_2_ NTs compared with the pristine In_2_O_3_ NFs (84.8 nm) (Figure 2c).

The intricate morphological and crystal characteristics of the WS_2_/In_2_O_3_ NFs were examined through the TEM and HRTEM. Figure 2d,e presents that the surface of In_2_O_3_ NFs is completely covered by WS_2_ NTs. In addition, the WS_2_/In_2_O_3_ NFs have a clear contrast between the dark interior and bright surface, confirming the WS_2_/In_2_O_3_ heterostructures. The representative HRTEM image (Figure 2f) reveals that the lattice fringe with a measured distance of 0.62 nm corresponds to the (002) planes of WS_2_ [25]. Since the WS_2_ nanosheets completely cover the surface of the In_2_O_3_ NFs and form a thick film, it is difficult to obtain the lattice fringes of the inner In_2_O_3_. The EDS elemental mappings analysis of WS_2_/In_2_O_3_ NFs in Figure 2g shows that In, O, S, and W elements are evenly distributed. Furthermore, Figure 2h presents the EDS spectrum of WS_2_/In_2_O_3_ NFs, in which all the elemental constituents including In, O, S, and W can be observed.

**Figure 3:**
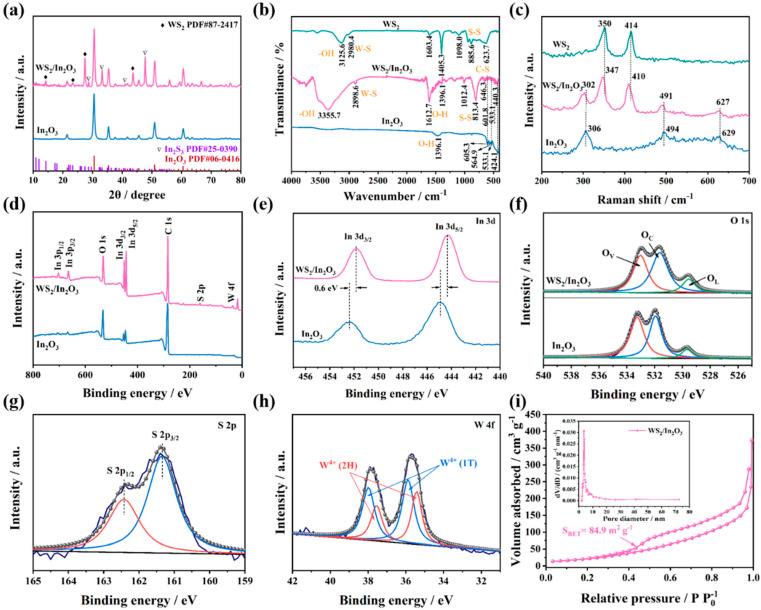
(**a**) XRD patterns of In_2_O_3_ and WS_2_/In_2_O_3_ NFs. (**b**) FT-IR spectra and (**c**) Raman spectra of pristine In_2_O_3_, WS_2_, and WS_2_/In_2_O_3_ NFs. XPS spectra of (**d**) survey, (**e**) In 3d, and (**f**) O 1s of In_2_O_3_ and WS_2_/In_2_O_3_ NFs; (**g**) S 2p, and (**h**) W 4f of WS_2_/In_2_O_3_ NFs. (**i**) N_2_ adsorption–desorption isotherms and pore-size distributions of WS_2_/In_2_O_3_ NFs.

The crystal structure and phase composition for pristine In_2_O_3_ and WS_2_/In_2_O_3_ NFs were investigated using XRD (Figure 3a). The peaks of pristine In_2_O_3_ correspond to the cubic In_2_O_3_ planes (JCPDS No. 06-0416) [26,27]. As for WS_2_/In_2_O_3_ heterojunctions, the typical peaks for WS_2_ at 14.39, 23.9, 28.15, and 44.94° are well indexed to WS_2_ (JCPDS No. 87-2417) [28], and peaks for In_2_S_3_ at 28.66, 33.22, 41.01, and 47.7 are indexed to In_2_S_3_ (JCPDS No. 25-0390) [29], can be observed alongside the prominent peaks attributed to In_2_O_3_, suggesting the successful formation of WS_2_/In_2_O_3_ heterojunctions. Furthermore, we have calculated the structural parameters of grain size and dislocation density for pristine In_2_O_3_ and WS_2_/In_2_O_3_ NFs, as shown in Table S1.

The FT-IR measurement was used to further explore the surface chemical information and vibrational modes of the chemical bonds in the pristine In_2_O_3_, WS_2_, and WS_2_/In_2_O_3_ composite (Figure 3b). The spectrum of pristine In_2_O_3_ with distinctive intense bands at 424.1533.1, 564.9, and 605.3 cm^−1^ were related to the In-O bond vibrations [30]. The band observed at 2980.4 cm^−1^ in WS_2_ is attributed to the bending vibrations of W-S bonds. Additionally, the vibrational band at 3125.6 cm^−1^ is associated with -OH, whereas the bands at 1098.0 and 623.7 cm^−1^ correspond to the vibrations of S-S and W-S bonds, respectively [17]. The characteristic peaks of WS_2_/In_2_O_3_ composite exhibit three vibrational peaks of In_2_O_3_ at 440.3, 533.1, and 601.8 cm^−1^, and three main peaks of WS_2_ at 2898.6, 1012.4, and 646.3 cm^−1^, suggesting the successful binding of In_2_O_3_ and WS_2_.

Figure 3c shows the Raman spectra of pristine In_2_O_3_, WS_2_, and WS_2_/In_2_O_3_ composite. In the case of the pristine In_2_O_3_, the Raman spectrum displays characteristic peaks at 306, 494, and 629 cm^−1^. These peaks correspond to the δ (InO_6_), In-O-In, and υ(InO_6_) of indium oxide structure, respectively [31]. The peaks of WS_2_ at 350 and 414 cm^−1^ corresponded to the E_2g_ and A_1g_ modes of WS_2_, respectively [32]. The Raman spectra of the WS_2_/In_2_O_3_ composite exhibited the distinctive peaks of both In_2_O_3_ and WS_2_, with no additional peaks observed, suggesting that the composite is made up exclusively of these two components. In addition, the characteristic peaks in the WS_2_/In_2_O_3_ composite show a noticeable red-shift compared to those of the pristine In_2_O_3_ and WS_2_, indicating the presence of lattice distortion or residual structural stress within the WS_2_/In_2_O_3_ [33].

XPS analysis facilitates a deeper understanding of the elemental composition present in the prepared samples. The full range spectra of pristine In_2_O_3_ and WS_2_/In_2_O_3_ are plotted in Figure 3d. The XPS survey spectrum of WS_2_/In_2_O_3_ presents the presence of main elements including In, O, S, W, and C. For In_2_O_3_, only binding energy peaks of In, O, and C can be observed. In Figure 3e, the XPS spectrum of In 3d for In_2_O_3_ exhibits two distinct peaks, one at a binding energy of 444.9 eV and another at 452.4 eV. These peaks are, respectively, ascribed to the In 3d_5/2_ and In 3d_3/2_, verifying the presence of In^3+^ [34,35]. Compared to In_2_O_3_, the In 3d peaks in WS_2_/In_2_O_3_ move to a lower binding energy (~0.6 eV), suggesting the presence of heterojunction, which significantly influences the chemical environment at the interface between In_2_O_3_ and WS_2_ [36,37]. The high-resolution O 1s spectra, as depicted in Figure 3f, can be deconvoluted into lattice oxygen (O_L_), oxygen vacancies (O_V_), and chemisorbed oxygen (O_C_) [29,38]. The area ratios of the oxygen fractions of samples are further listed in Table S2. The contents of O_V_ in pristine In_2_O_3_ and WS_2_/In_2_O_3_ NFs are 40.8% and 48.4%, respectively. The increase in O_V_ content of WS_2_/In_2_O_3_ NFs is likely a result of the lattice distortions due to the creation of heterojunctions. The S 2p spectrum of WS_2_/In_2_O_3_ heterojunctions is depicted in Figure 3g. Characteristic peaks observed at 162.4 and 161.3 eV in the XPS spectrum are specifically attributed to the binding energies associated with the S 2p_1/2_ and S 2p_3/2_, respectively [39,40]. The spectrum W 4f of WS_2_/In_2_O_3_ in Figure 3h can be fitted into two components and the peaks located at 35.6 and 37.8 eV were indexed with W 4f_5/2_ and W 4f_7/2_. Both peaks comprise W^4+^ (1T) and W^4+^ (2H), suggesting that tungsten is present in the +4 oxidation state [41,42].

The nitrogen adsorption–desorption isotherms, along with the pore size distribution curves for the pristine In_2_O_3_ and the WS_2_/In_2_O_3_ composite, are depicted in Figure S1 and Figure 3i. For the pristine In_2_O_3_ and WS_2_/In_2_O_3_ NFs, the N_2_ adsorption/desorption isotherms exhibit the characteristic type IV isotherm, accompanied by a H3 hysteretic loop, suggesting the presence of mesoporous structure within both samples. The pore size for pristine In_2_O_3_ and WS_2_/In_2_O_3_ NFs (inset in Figure S1 and Figure 3i) are about 2.7 and 3.5 nm, respectively. And the pore volumes of WS_2_/In_2_O_3_ NFs are higher than those of pristine In_2_O_3_ NFs. Meanwhile, the WS_2_/In_2_O_3_ NFs possess a significantly larger specific surface area of 84.9 m^2^ g^−1^. This value is markedly superior to that of the pristine In_2_O_3_, which has a specific surface area of only 9.6 m^2^ g^−1^. The integration of WS_2_ NTs onto the surface of In_2_O_3_ NFs has led to a notable increase in the specific surface area and a corresponding rise in the pore volume. This structural modification is advantageous, as it promotes the diffusion and adsorption processes of O_2_ and HCHO molecules within the sensing material, thereby enhancing the material’s sensitivity in detecting the target gases [7].

### 3.2. Gas Sensing Performance

**Figure 4:**
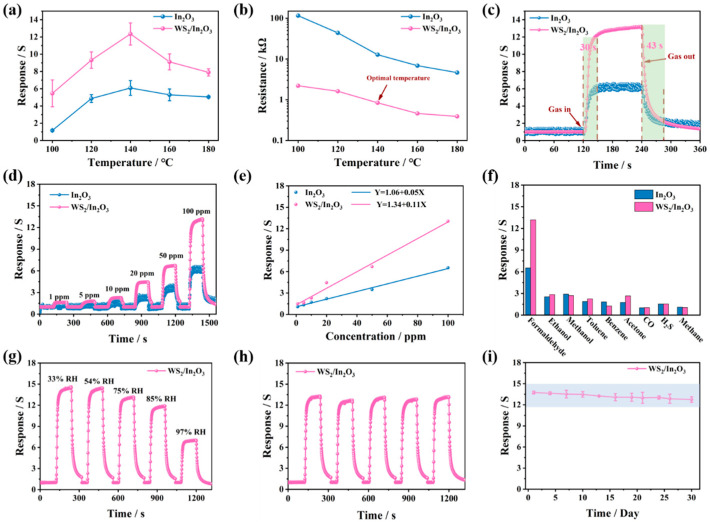
Sensing properties measurements for pristine In_2_O_3_ and WS_2_/In_2_O_3_ sensors at 140 °C. (**a**) Responses toward HCHO (100 ppm) under operating temperatures from 100 to 180 °C; (**b**) base resistance in air of the two sensors at varied operating temperatures; (**c**) the dynamic sensing transients to 100 ppm HCHO; (**d**) the dynamic response and recovery curves of sensors to different concentrations of HCHO; (**e**) fitting linearity curves of the concentration of sensors vs. the response value; (**f**) cross-responses of two sensors for detecting 100 ppm of various gases; (**g**) dynamic response curves of the WS_2_/In_2_O_3_ sensor to 100 ppm HCHO in different humid conditions; (**h**) the reproducibility of the WS_2_/In_2_O_3_ sensor toward 100 ppm HCHO; (**i**) response stable characteristics of the WS_2_/In_2_O_3_ sensor to 100 ppm HCHO.

For the optimization of the sensor operating condition, the operating temperature is considered an essential factor. The corresponding responses of pristine In_2_O_3_ and WS_2_/In_2_O_3_ sensors exposed to 100 ppm HCHO were recorded at different operating temperatures, as displayed in Figure 4a. Within the temperature interval from 100 to 140 °C, there was a positive correlation between the operating temperature and the sensor responses for both sensors. Upon reaching an operating temperature of 140 °C, the responses of the sensors reached the maximum values of 6.2 (In_2_O_3_) and 12.6 (WS_2_/In_2_O_3_), respectively. Beyond this temperature, a decline in the responses was observed. Thus, the optimal operating temperature of the pristine In_2_O_3_ and WS_2_/In_2_O_3_ sensors is 140 °C and all further gas sensing investigations were performed at 140 °C. In addition, thermal gravimetric (TG) analysis was conducted to understand the thermal decomposition behavior of In_2_O_3_ and WS_2_/In_2_O_3_ NFs (Figure S2). The results show that the In_2_O_3_ and WS_2_/In_2_O_3_ NFs had good thermal stability. Figure 4b presents the baseline resistance (R_a_) of the sensors measured in ambient air across a range of operating temperatures from 100 to 140 °C. The experimental findings indicate a decrease in the electrical resistance (R_a_) for both sensors as the temperature elevates. This phenomenon can be ascribed to the inherent properties of metal oxide semiconductors [43].

The response and recovery curves to 100 ppm HCHO of both sensors at 140 °C were depicted in Figure 4c and Figure S3. The response/recovery times (τ_res_./τ_rec_.) of pristine In_2_O_3_ and WS_2_/In_2_O_3_ sensors were calculated to be 23/50 s and 30/43 s, respectively. The recovery times were longer than that of the response time, which is attributed to the relatively slower desorption kinetics of the by-products involved in the process [18].

The responses comparison for the pristine In_2_O_3_ and WS_2_/In_2_O_3_ sensors to different concentrations (1–100 ppm) of HCHO at 140 °C is shown in Figure 4d. With the rise in concentration, the response correspondingly increases. The response values of pristine In_2_O_3_ sensor to 1, 5, 10, 20, 50, and 100 ppm HCHO are 1.2, 1.4, 1.9, 2.6, 4.1, and 6.2, respectively, whereas the WS_2_/In_2_O_3_ sensor’s response values are 1.7, 1.9, 2.3, 4.5, 6.7, and 12.6 for the same HCHO concentrations, indicating a notable improvement in the response of the sensor. Additionally, Figure 4e presents the linear calibration curves associated with the sensor’s response. The response values of both sensors demonstrate a good linear correlation with gas concentration [5].

The ability to selectively identify target gases is paramount in establishing the sensing properties of a gas sensor for effective performance within a complex atmosphere [6]. Figure 4f shows the selectivity of pristine In_2_O_3_ and WS_2_/In_2_O_3_ sensors for nine different gases at a concentration of 100 ppm. The obtained results indicated that the WS_2_/In_2_O_3_ sensor were more sensitive and selective toward HCHO compared to other potential interfering gases. The bond dissociation energy of the target gas molecules is a critical factor that influences the sensor’s selectivity. As detailed in Table S3, the bond dissociation energy for the HCHO molecule (364 kJ/mol) is lower than that of other gases, demonstrating that HCHO is more likely to react with adsorbed oxygen molecules than other gas molecules. Consequently, the WS_2_/In_2_O_3_ sensor exhibits a higher sensitivity to HCHO in comparison to other gases that possess higher bond dissociation energies. In addition, the response of the WS_2_/In_2_O_3_ sensor is higher than that of the pristine In_2_O_3_ sensor, demonstrating its excellent selectivity toward HCHO.

Additionally, the impact of humidity on the sensing performance of the WS_2_/In_2_O_3_ sensor was assessed under varying water vapor conditions, ranging from 33% to 97% relative humidity (RH) in Figure 4g. As the relative humidity rises to 85% RH, the WS_2_/In_2_O_3_ sensor’s response value experiences a minor reduction, amounting to 18%. When the humidity reaches 97% RH, the response value drops to 7.1. This reduction is caused by the competition for active surface sites on the sensing material, where water molecules take up spaces that are crucial for interaction with the target gas [14].

Figure 4h presents the cyclic response and recovery curves of the WS_2_/In_2_O_3_ sensor when exposed to 100 ppm of HCHO at an operating temperature of 140 °C. The sensor demonstrates five consecutive cycles with only minor fluctuations in the response value, and the response and recovery times remain consistent. This consistency indicates the sensor’s excellent reproducibility. Furthermore, Figure 4i exhibits the long-term stability assessment of the WS_2_/In_2_O_3_ sensor over 30 days. The response value shows minimal variation throughout this duration, indicating the sensor’s remarkable stability over an extended period. Additionally, a comparative analysis of the sensing capabilities of the WS_2_/In_2_O_3_ sensor against those of other HCHO sensors is detailed in Table S4. The high sensitivity and low operating temperature of the WS_2_/In_2_O_3_ sensor greatly enhance the competitiveness of this research in the field of gas sensing technology.

### 3.3. Gas Sensing Mechanism

As a classic n-type In_2_O_3_, when it is exposed to an air environment, oxygen molecules will adhere to the surface of the In_2_O_3_. Subsequently, the interaction with these molecules leads to the capture of electrons from the conduction band, which in turn results in the formation of various species of oxygen anions (O_2_^−^, O^−^, and O^2−^) at different operating temperatures (150 °C or less, between 150 and 400 °C, or more than 400 °C). Simultaneously, the formation of an electron depletion layer on the In_2_O_3_ surface leads to a consequent rise in electrical resistance (R_a_) [44]. In our study, the optimal operating temperature determined for the sensor is 140 °C. At this temperature, the adsorbed oxygen species on the sensor’s surface predominantly exist in the form of oxygen ions with a charge of O_2_^−^ (Equation (2)). Furthermore, upon exposure to reducing HCHO gas, the HCHO molecules interact with O_2_^−^, transferring electrons to the conduction band of In_2_O_3_. This electron transfer effectively reduces the sensor’s resistance (R_g_) [45]. The interactions involving adsorbed oxygen and HCHO are detailed in the subsequent Equations (1) and (3) [46,47]:

O_2_ (gas) → O_2_ (ads) (1)

O_2_ (ads) + e^−^ → 2O_2_^−^ (ads) (2)

HCHO (gas) + 2O_2_^−^ (ads) → CO_2_ (gas)+ H_2_O (gas) +2e^−^ (3)

**Figure 5:**
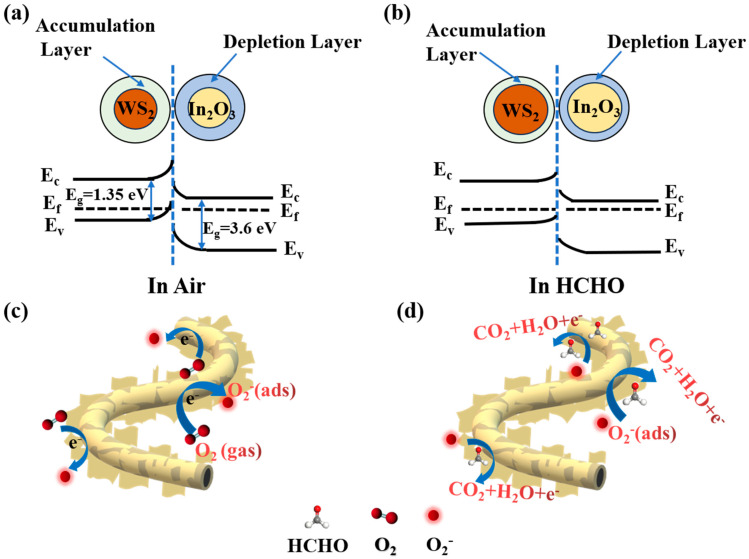
(**a**,**b**) The energy band diagram and (**c**,**d**) the schematic of the gas sensing mechanism of WS_2_/In_2_O_3_ NFs.

The schematic sensing mechanism of WS_2_/In_2_O_3_ NFs is shown in Figure 5. The improved gas sensing performance of WS_2_/In_2_O_3_ compared to the pristine In_2_O_3_ NFs sensor is predominantly attributed to the following two key factors:

Firstly, the p-n heterojunction between WS_2_ and In_2_O_3_. Figure 5a,b present the energy band diagrams of the WS_2_/In_2_O_3_. When the p-type properties of WS_2_ are in close contact with the n-type properties of In_2_O_3_, the Fermi levels should be equal, which will lead to the transfer of electrons from In_2_O_3_ to WS_2_, while the holes move in the opposite direction [19,48,49]. In Figure 5a, the electron depletion layers (EDL) and the electron accumulation layer (EAL) form at the interface of In_2_O_3_ and WS_2_ in an air environment, respectively. In Figure 5b, upon exposure to HCHO gas, the HCHO molecules chemically interact with the O_2_^−^ that are adsorbed on the surface. This interaction breaks the equilibrium state of the intrinsic electric field under air conditions. As a result, the trapped electrons are released back to the In_2_O_3_, leading to an enhancement of electron density on In_2_O_3_ and a decrease in the electrical resistance of the sensor [50]. Consequently, the sensitivity of the WS_2_/In_2_O_3_ heterojunction to HCHO gas is significantly amplified.

Secondly, the increased content of O_V_ offers a greater quantity of reactive sites for gs adsorption and contributes to the enhanced response of the WS_2_/In_2_O_3_ sensor. Throughout the gas detection process, oxygen vacancies (O_V_) frequently serve as sites for the adsorption and reaction of gases, as they possess a lower binding energy [51,52]. In this study, XPS analysis reveals that the WS_2_/In_2_O_3_ heterojunction contains a higher concentration of O_V_ compared to the pristine In_2_O_3_. This increased O_V_ content leads to greater adsorption of oxygen and HCHO on the WS_2_/In_2_O_3_ surface, which in turn facilitates the gas sensing reactions.

Accordingly, the improved sensing performance of WS_2_/In_2_O_3_ heterojunction is because of the synergistic effect of the increased charge transfer and the presence of more sites for adsorption.

## 4. Conclusions

In conclusion, 2D/1D heterostructure WS_2_/In_2_O_3_ NFs were fabricated via the electrospinning process, followed by hydrothermal treatment. The WS_2_/In_2_O_3_ NFs sensor shows an excellent response of 12.6 toward HCHO (100 ppm) at 140 °C along with a short response/recovery time (30/43 s). Furthermore, the sensor possesses exceptional selectivity and long-term stability. The improved detection capabilities for HCHO gas can be ascribed to the enhanced charge transfer at the WS_2_/In_2_O_3_ heterojunctions interfaces as well as the increased adsorption sites. The combination of 2D WS_2_ nanosheets with the 1D In_2_O_3_ nanofibers has been validated as a potent approach for the synthesis of sensitive material for efficient HCHO detection.

## Data Availability

The data that have been used are confidential.

## Supplementary Materials

The following supporting information can be downloaded at: https://www.mdpi.com/article/10.3390/nano14211702/s1, Text S1; Figure S1: N_2_ adsorption-desorption isotherms and pore-size distributions of pristine In_2_O_3_; Figure S2: TGA analysis of (a) In_2_O_3_ and (b) WS_2_/In_2_O_3_ NFs; Figure S3: Response and recovery characteristics of sensors exposure to HCHO (100 ppm); Table S1: Structural parameters for pristine In_2_O_3_ and WS_2_/In_2_O_3_ NFs by considering the crystal plane from their XRD patterns; Table S2: The relative percentages of three different oxygen species for pristine In_2_O_3_, and WS_2_/In_2_O_3_ NFs; Table S3: The molecular structure and the bond dissociation energies of various gases molecules; Table S4: Comparison of HCHO gas sensing performance with other gas sensors. References [53,54,55,56,57,58,59] are cited in the supplementary materials.
