# Supplementary material for: Hierarchical Heterojunctions of Metal Sulfide WS2 Nanosheets/Metal Oxide In2O3 Nanofibers for an Efficient Detection of Formaldehyde
Source: Nanomaterials (Basel). 2024 Oct 24;14(21):1702. doi: 10.3390/nano14211702 (PMC11547418; doi:10.3390/nano14211702)
Supplement: Supplementary file 1 [file nanomaterials-14-01702-s001.zip › nanomaterials-3261411-supplementary.pdf]

Text. S1

Initially, air from a reference cylinder was steadily fed into the gas distribution chamber at a flow rate of 50 milliliters per minute (mL/min) to displace the existing air within the chamber, a process that continued for a duration of 10 minutes. Upon ceasing the air supply, a measured volume Q of a 37% HCHO solution was extracted using a microsyringe, subsequently injected into the evaporator located within the gas distribution chamber, and subjected to heating for complete evaporation. Ultimately, the resultant HCHO vapor was thoroughly mixed with the reference air by the action of an internal fan, ensuring a homogeneous mixture. Specifically, acetone, benzene, toluene, methanol, ethanol, and formaldehyde vapors were obtained by evaporating acetone (≧99.5%), benzene (≧ 99.5%), toluene (≧ 99.5%), methanol (≧ 99.5%), ethanol (≧ 99.7%), and formaldehyde (37%) solution, respectively. The operating temperature of the sensor was controlled by regulating the voltage of the heating wire. The different concentrations of test gases were prepared based on the static gas distribution method. A certain volume Q of gas or liquid was injected into a testing chamber, and the evaporation system quickly volatilized the injected liquid into vapor. The volume Q can be determined by the following equation:

$$Q = \frac{V \times C \times M}{22.4 \times d \times \rho} \times 10^{-9} \times \frac{273 + T_R}{273 + T_B} \quad \text{(S1)}$$

Where V, C, M, d, ρ, T_R, and T_B are the test chamber volume (mL), vapor concentration (ppm), liquid molecular mass (g), liquid density (g /cm^3), liquid purity, environmental temperature (°C), and temperature in the testing chamber (°C), respectively. The stable resistance value of the sensor in air (or test gas) is labeled as R_a (or R_g). The sensitivity is defined as S = R_a/R_g. The response and recovery times are calculated based on the time from its response to reach 90% variation of the total resistance.

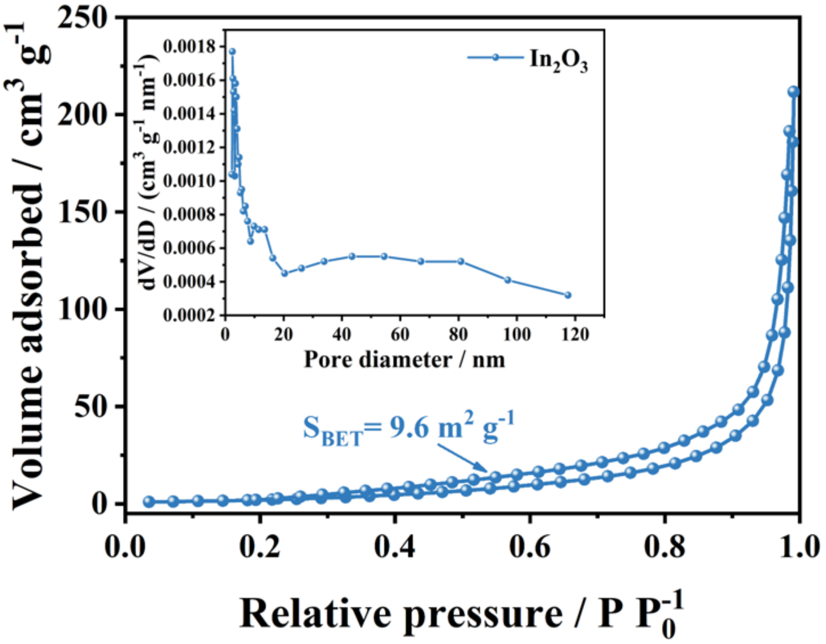

Figure S1. N_2 adsorption-desorption isotherms and pore-size distributions of pristine In_2O_3

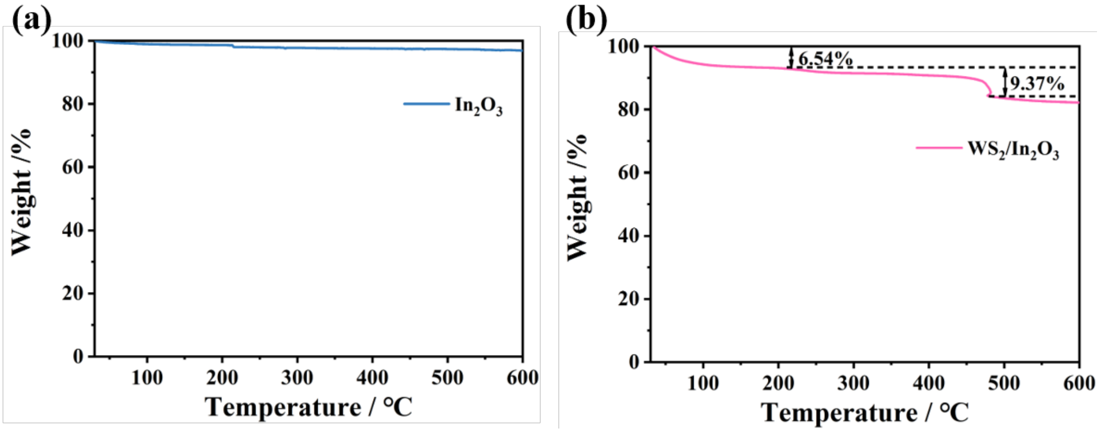

Figure S2. TGA analysis of (a) In_2O_3 and (b) WS_2/In_2O_3 NFs.

Thermal gravimetric (TG) analysis was conducted to understand the thermal decomposition behavior of In_2O_3 and WS_2/In_2O_3 NFs (Fig. S2). The In_2O_3 NFs exhibit high-heat thermal stability up to 600 °C except for the initial mass loss (Fig. S2a). In the case of WS_2/In_2O_3 NFs, it initially exhibits low weight loss (6.54 %) from room temperature to 210 °C, which corresponds to the release of guest species such as moisture/water molecule. The second TG mass loss of 9.37 % between 210 °C and 480 °C was caused by the detachment/elimination of the oxygen-containing functional groups (Fig. S2b)[1]. The TGA study shows that the In_2O_3 and WS_2/In_2O_3 NFs had good thermal stability.

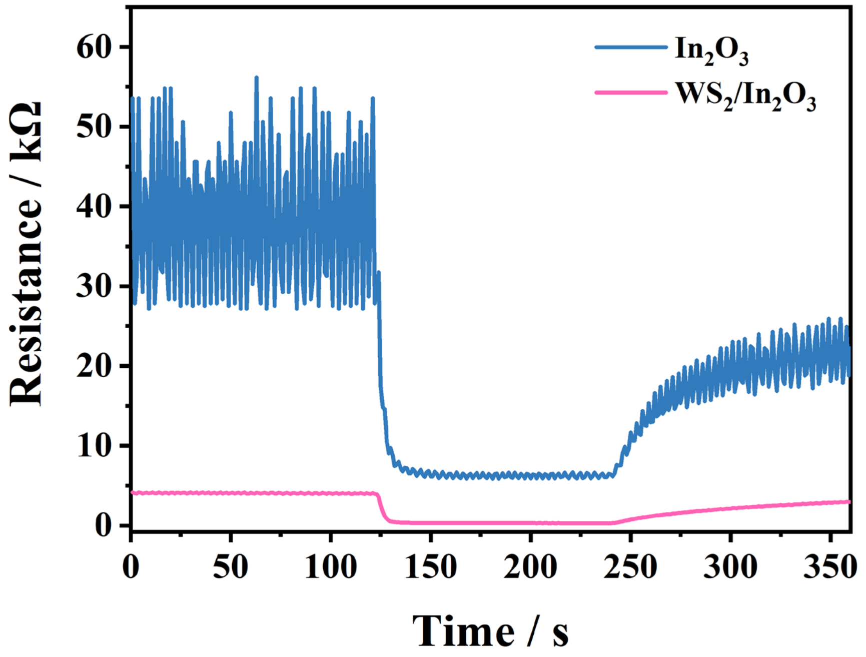

Figure S3. Response and recovery characteristics of sensors exposure to HCHO (100 ppm)

Table S1 Structural parameters for pristine In_2O_3 and WS_2/In_2O_3 NFs by considering the crystal plane from their XRD patterns.

Materials	2Theta (degree)	FWHM (degree)	Grain size (nm)	Dislocation density (nm^{-2})
In_2O_3	30.513	0.494	16.67	0.0035
WS_2/In_2O_3	30.544	0.519	15.86	0.0040

The structural parameters of pristine In_2O_3 and WS_2/In_2O_3 NFs have been taken into account by considering the most intense peak (222) according to the XRD patterns as shown in Figure 4a. These structural parameters were calculated using Scherrer's formula and Bragg's law as follows and shown in Table S1:

$$D = 0.9\lambda/\beta\cos\theta \qquad (1)$$

$$\delta = 1/D^2 \qquad (2)$$

where λ represents the incident wavelength of X-ray in nm, β represents the full width at half maximum (FWHM) in radians, θ represents the Bragg angle in radians, D represents the grain size in nm, and δ represents the dislocation density in nm^{-2}. The calculated grain sizes and FWHM values along with dislocation density of pristine In_2O_3 and WS_2/In_2O_3 NFs are shown in Table S1. The FWHM values were found as 0.494° and 0.519° for pristine In_2O_3 and WS_2/In_2O_3 NFs, respectively. And the decrease in crystallite size and increment dislocation density for WS_2/In_2O_3 NFs is attributed to the formation of WS_2/In_2O_3 heterojunctions, which caused structural disordering and increased dislocation density [2].

Table S2. The relative percentages of three different oxygen species for pristine In_2O_3, and WS_2/In_2O_3 NFs.

Sample	Oxygen Species	Relative Percentage (%)
pristine In_2O_3	O_L	10.9
	O_v	40.8
	O_c	48.3
WS_2/In_2O_3	O_L	12.5
	O_v	48.4
	O_c	39.1

Table S3. The molecular structure and the bond dissociation energies of various gases molecules

Gas type	Formaldehyde	Methane	H_2S	Ethanol	Methanol	Benzene	Toluene	Acetone	CO
Structural	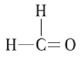			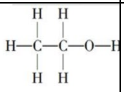	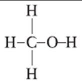	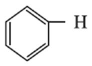	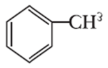	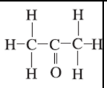	
Bond	H-CHO	C-H	H-SH	H-OCH_2CH_3	H-OCH_3<br>H-CH_2<br>H-CH	H-C_6H_5	CH_3-C_6H_5	H-CH_2COCH_3	C-O
Bond dissociation energy ($kJ\ mol^{-1}$)	364	416	381	436	436.8<br>473<br>452	431	389	393	1071

Table S4. Comparison of HCHO gas sensing performance with other gas sensors

Materials	Temperature / °C	Concentration / ppm	Response	t_{res}/t_{rec} / s	Ref.
SnO_2/Fe_2O_3	220	20	4.5	9 / 34	[3]
Co_3O_4/ZnO	120	10	6.17	6 / 4	[4]
$Au-In_2O_3$	240	100	37	3 / 8	[5]
Co_3O_4-ZnO core-shell	220	100	5.3	6 / 9	[6]
Co_3O_4	170	100	12	46 / 98	[7]
$Ni-In_2O_3/WS_2$	25	5	-	76/ 123	[8]
WS_2/In_2O_3 NFs	140	100	12.6	30 / 43	**This work**

References

[1] J.R. Xavier, R.B. J, Evaluation of reduced graphene oxide/WO3/WS2 hybrids for high performance supercapacitor electrode, J. Alloys Compd., 947 (2023).

[2] A.K. Singh, C.-C. Yen, D.-S. Wuu, Structural and photodetector characteristics of Zn and Al incorporated ZnGa2O4 films via co-sputtering, Results in Physics, 33 (2022).

[3] C. Lou, Q. Huang, Z. Li, G. Lei, X. Liu, J. Zhang, Fe_2O_3-sensitized SnO_2 nanosheets via atomic layer deposition for sensitive formaldehyde detection, Sens. Actuators, B, 345 (2021).

[4] J. Sun, L. Sun, S. Bai, H. Fu, J. Guo, Y. Feng, R. Luo, D. Li, A. Chen, Pyrolyzing Co/Zn bimetallic organic framework to form p-n heterojunction of Co3O4/ZnO for detection of formaldehyde, Sens. Actuators, B, 285 (2019) 291-301.

[5] S. Zhang, P. Song, J. Li, J. Zhang, Z. Yang, Q. Wang, Facile approach to prepare hierarchical Au-loaded In_2O_3 porous nanocubes and their enhanced sensing performance towards formaldehyde, Sens. Actuators, B, 241 (2017) 1130-1138.

[6] X. Gao, F. Li, R. Wang, T. Zhang, A formaldehyde sensor: Significant role of p-n heterojunction in gas-sensitive core-shell nanofibers, Sens. Actuators, B, 258 (2018) 1230-1241.

[7] W. Zhou, Y.P. Wu, J. Zhao, W.W. Dong, X.Q. Qiao, D.F. Hou, X. Bu, D.S. Li, Efficient Gas-Sensing for Formaldehyde with 3D Hierarchical Co3O4 Derived from Co5-Based MOF Microcrystals, Inorg. Chem., 56 (2017) 14111-14117.

[8] D.Z. Zhang, Y.H. Cao, Z.M. Yang, J.F. Wu, Nanoheterostructure construction and DFT study of Ni-doped In_2O_3 nanocubes/WS_2 hexagon nanosheets for formaldehyde sensing at room temperature, ACS Appl. Mater. Interfaces, 12 (2020) 11979-11989.
